# Enabling solid sample analysis in liquid spectrophotometers with a 3D-printed cuvette

**DOI:** 10.1038/s41598-025-88611-2

**Published:** 2025-02-20

**Authors:** Jacques Doumani, Henry Mansfield, Andrey Baydin, Somesh Sasmal, Mario El Tahchi, Weilu Gao, Junichiro Kono

**Affiliations:** 1 Department of Electrical and Computer Engineering, Rice University, Houston, TX 77005 USA; 2 Rice Advanced Materials Institute, Rice University, Houston, TX 77005 USA; 3 Smalley-Curl Institute, Rice University, Houston, TX 77005 USA; 4 Department of Materials Science and NanoEngineering, Rice University, Houston, TX 77005 USA; 5 Department of Physics, Lebanese University, Jdeidet, 90656 Lebanon; 6 Laboratory of Biomaterials and Intelligent Materials, Lebanese University, Jdeidet, 90656 Lebanon; 7 Department of Electrical and Computer Engineering, The University of Utah, Salt Lake City, UT 84112 USA; 8 Carbon Hub, Rice University, Houston, TX 77005 USA; 9 Department of Physics and Astronomy, Rice University, Houston, TX 77005 USA

**Keywords:** Cuvette, Spectrophotometry, Circular Dichroism, Carbon Nanotubes, Circular dichroism, Spectrophotometry, Spectrophotometry

## Abstract

Most commercial systems for ultraviolet-visible (UV–VIS), Fourier-transform infrared, circular dichroism (CD), and fluorescence spectroscopies are designed for measurement of liquid samples. Moreover, adapters enabling the measurement of solid samples are expensive or unavailable for most commercial instruments. Consequently, there is a significant need for solid sample adapters that enable measurement of both liquid and solid samples with a single system. Here, we report two versions of a solid sample adapter cuvette that can be used in most commercial spectroscopy instruments designed for transmission measurement of liquid samples. One version is designed for techniques that do not require changing the sample orientation, and the other allows easy sample rotation. We successfully fabricated these cuvettes by 3D printing with both fused deposition modeling and stereolithography and demonstrated how they enable us to study the optical properties of macroscopic films of aligned carbon nanotubes by performing UV–VIS and CD spectroscopy measurements with the cuvettes. These 3D printed cuvettes and their implementation will help enable a wide range of experiments at a low cost.

**Supplementary Information:**

The online version contains supplementary material available at 10.1038/s41598-025-88611-2.

## Introduction

Optical spectroscopy is critical for materials characterization and the study of interaction between matter and electromagnetic radiation. Techniques thereof include infrared spectroscopy, which is used to study the vibrations of chemical bonds and identify functional groups in compounds^1^, absorption and fluorescence spectroscopies, which are used to study electronic transitions in molecules and condensed matter systems^2,3^, and circular dichroism (CD) spectroscopy, which is used to probe the chirality of molecules and materials^4^. Holders for liquid samples, or cuvettes, are essential components in these techniques as they ensure the accuracy and reliability of the measurements. Cuvettes and other accessories also have the ability to accommodate various sample types, enhancing the range of experiments possible with an instrument.

Currently, ultraviolet-visible (UV–VIS) spectrophotometers with various types of cuvettes are commercially available and are situated in chemistry, physics, engineering, and materials science laboratories all over the world. However, there are many experiments that study solid samples for which sample holders or adapters are expensive or do not exist. Thus, solid-state physicists and materials scientists are often required to purchase new spectrophotometer systems or build complex setups to enable their experiments. 3D printing cuvettes optimized for specific samples is a cheap and flexible alternative to these options.

As 3D printing technology has developed and become more widely available, there have been reports regarding 3D printed cuvettes and cuvette adapters customized to allow analysis of samples with specific sizes, compositions, and optical properties^5–13^. For example, a universal cuvette adapter that extends existing UV–VIS spectrophotometer capabilities^8^, a universal design for a photometric-fluorometric, UV–VIS compatible, 3D-printed flow-through cuvette^9^, and an adapter for solid fluorescence measurements^12^ have been reported. Nevertheless, there remains a lack of a solid sample cuvette that can be used in most commercial spectroscopy systems designed for liquid samples. Furthermore, to the best of our knowledge, there does not exist a cuvette that facilitates measurement of solid samples at different orientations for use in CD spectroscopy.

Here, we report on the design and fabrication of two 3D printed cuvettes that can be used to analyze solid samples in UV–VIS spectrophotometers and CD spectrometers designed for liquid samples. One version, the stationary cuvette, is designed for techniques that do not require changing the sample orientation, and the other, the rotating cuvette, allows easy sample rotation, facilitating techniques such as CD. Both cuvettes can be printed quickly, cost <$1, and are easily modifiable for experiments outside of those presented in this article.

We demonstrated the successful use of these cuvettes in experiments on carbon nanotube (CNT) films in instruments designed for liquid samples. CNTs have distinct spectral features throughout the UV–VIS range^14^, and strong anisotropy when in the form of an aligned film^15^, making CNT films ideal solid samples in which to demonstrate the functionality of our cuvettes. We obtained accurate UV–VIS attenuation spectra measured with the stationary cuvette. We also demonstrated the rotating cuvette’s ability to yield accurate CD measurements through precise sample rotation. Our cuvettes enable us to find the alignment axis and the nematic order parameter of CNT thin films, and measure the effects of liquid and solid environments on CNT excitons.

While we demonstrate the cuvettes’ functionality with CNT films, our cuvettes are easily customizable to support transmission measurements of other solid sample geometries and compositions.

## Results

**Fig. 1:**
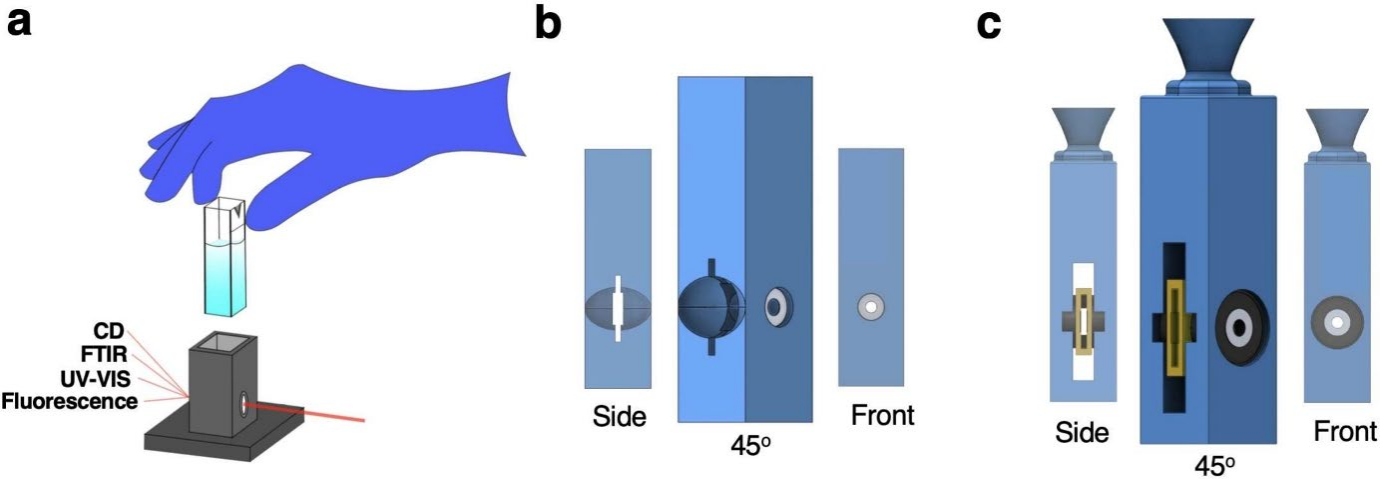
Solid sample cuvette design. (**a**) Liquid sample holders, or cuvettes, are required for most spectroscopy experiments including Fourier transform infrared (FTIR), ultraviolet-visible (UV–VIS), fluorescence, and circular dichroism (CD) spectroscopies. (**b**) The stationary cuvette can be used in place of liquid sample cuvettes to enable solid sample measurements that only require measurement at one orientation, such as UV–VIS, FTIR, and fluorescence spectroscopies. The cutout facilitates easy sample insertion and removal, and the aperture system ensures that light doesn’t interact with the cuvette’s assembly materials. (**c**) The rotating cuvette can be used in place of liquid sample cuvettes to enable solid sample measurements that require multiple orientations, such as CD spectroscopy. The cuvette uses a sample container (shown in yellow) that can rotate about the beam axis using apertures made from ball bearings.

Cuvettes for holding liquid samples are integral components in almost all UV–VIS spectrophotometers and CD spectrometers (Fig. 1a). However, different types of commercial cuvettes, accessories, or even different instruments are often required to study solid samples, introducing additional research costs. To address this issue, we designed two types of cuvettes for solid samples that are compatible with commercial instruments designed for liquid-type samples (Fig. 1b,c).

The cuvette shown in Fig. 1b is designed for measurements that only require one sample orientation. A solid sample can be inserted into the slot visible in the side view, while a cutout facilitates easy sample insertion and removal. The cuvette uses a simple aperture system of two washers to ensure light only passes through the sample.

The second of these cuvettes, depicted in Fig. 1c, is designed for measurements requiring rotation about the optical axis, such as CD measurement of solid samples. This cuvette features a more complex aperture system, consisting of two bearings and two washers. A solid sample is placed into the yellow pocket, and the bearing-washer apertures allow the yellow pocket to rotate about the beam axis even when the sample is inserted. This symmetric and high-precision design guarantees that the same sample area is measured irrespective of the azimuthal orientation.

The stationary cuvette (Fig. 1b) measures 45$\times$12.5$\times$12.5 mm$^3$ while the rotating cuvette (Fig. 1c) measures 55$\times$12.5$\times$12.5 mm$^3$. Both cuvettes feature apertures centered at a height of 15 mm and are optimized to hold samples of dimensions 10$\times$10$\times$1 mm$^3$. Parts and equipment used for fabrication are listed in SI Table 1. Customizable 3D models of the cuvettes and slicing and printing information for both fused deposition modeling (FDM) and stereolithography (SLA) 3D printing are attached in the Supporting Information.

**Fig. 2:**
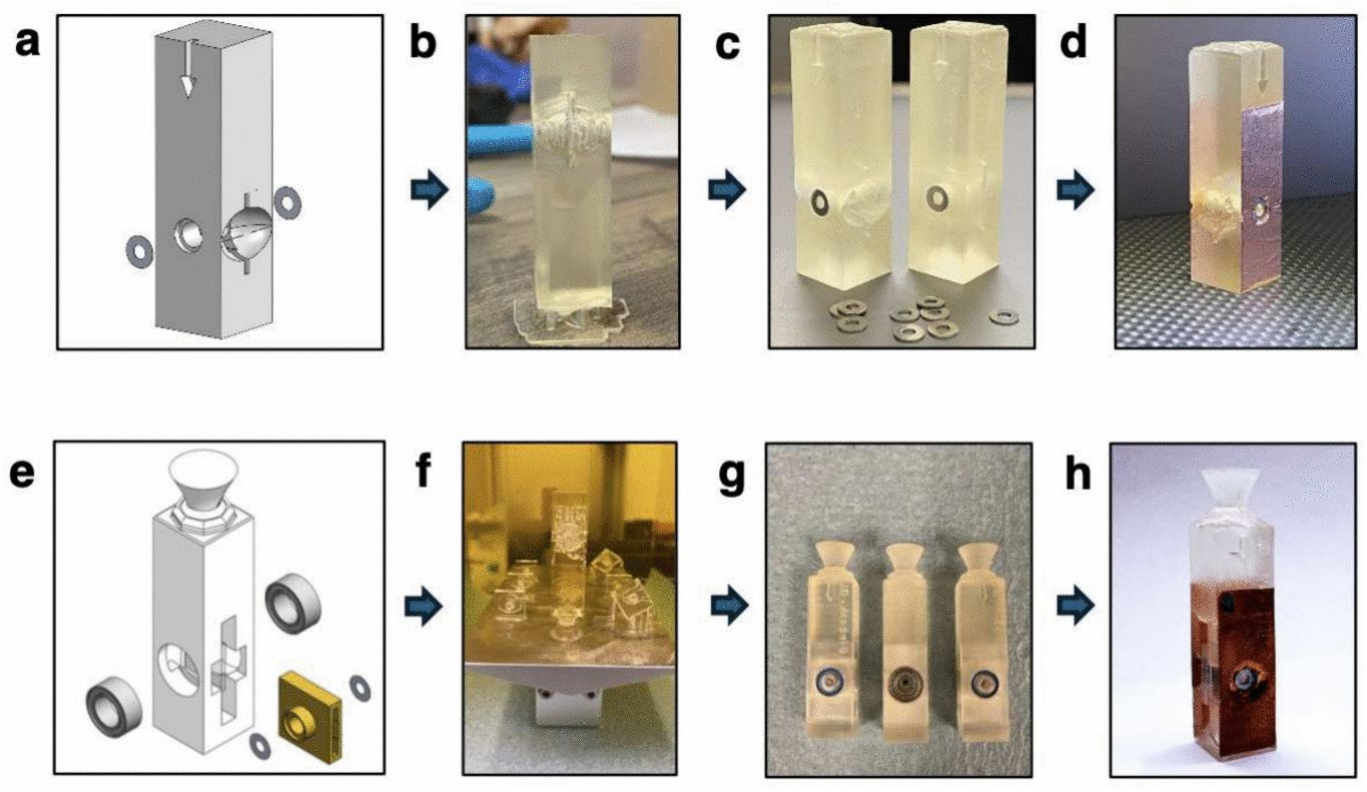
SLA cuvette fabrication. Throughout the figure the top images are of the stationary cuvette, and the bottom images are of rotating cuvette. (**a**,**e**) Screenshots of cuvette 3D models. Both 3D models were designed in Solidworks, and imported to slicing software to control printing. (**b**,**f**) Photograph of SLA 3D printed cuvettes and sample pockets. All prints required supports and were printed in a bottom down configuration to prevent collapse. Supports and excess resin were removed and parts were placed in a bath sonicator before UV curing and cuvette assembly. (**c**,**g**) Photograph of assembled cuvette. SLA cuvettes were assembled before UV curing in order to lock the aperture systems in place before full resin solidification. Stationary cuvettes required two washers, and rotating cuvettes required two bearings, two washers, and a 3D printed sample pocket to enable sample rotation. (**d**,**h**) Photograph of completed cuvettes. Cuvettes were finished with copper tape to prevent excess light transmission through gaps in the aperture systems.

Cuvette fabrication processes with SLA 3D printing are shown in Fig. 2 for both the stationary and rotating models. Designs were created using SolidWorks (Fig. 2a,e) and were sliced and printed using Chitubox basic software and Anycubic Photon Workshop. The files were printed in various positions, and it was determined that both parts were best oriented in a vertical configuration, where the top of the cuvette is positioned downward (Fig. 2b,f). The printing process took five hours for SLA printing with a resin cost of <$1. Following SLA printing, supports were removed, and the parts were cleaned and placed in a bath sonicator to remove excess resin. In the stationary cuvette, washers were used as apertures, and in the rotating cuvette, a system of washers and bearings were used as apertures (Fig. 2c,g). After assembly, SLA printed cuvettes were cured to keep the aperture components in place. Finally, copper tape was applied to both sides of the cuvette (Fig. 2d,h) to prevent transmission through the cuvette body or any gaps in the aperture systems.

Although not shown, both parts were replicated using FDM printing. FDM cuvettes were also designed with Solidworks but were sliced and printed using Cura software. FDM parts were also printed in a vertical configuration with the bottom face of the cuvette positioned downward. Printing took approximately two hours and used <$1 of filament. FDM printed parts did not require bath sonication or UV curing, but copper tape was applied before the completed cuvettes were used. More detailed information on both SLA and FDM 3D printing and cuvette assembly can be found in the Supporting Information.

**Fig. 3:**
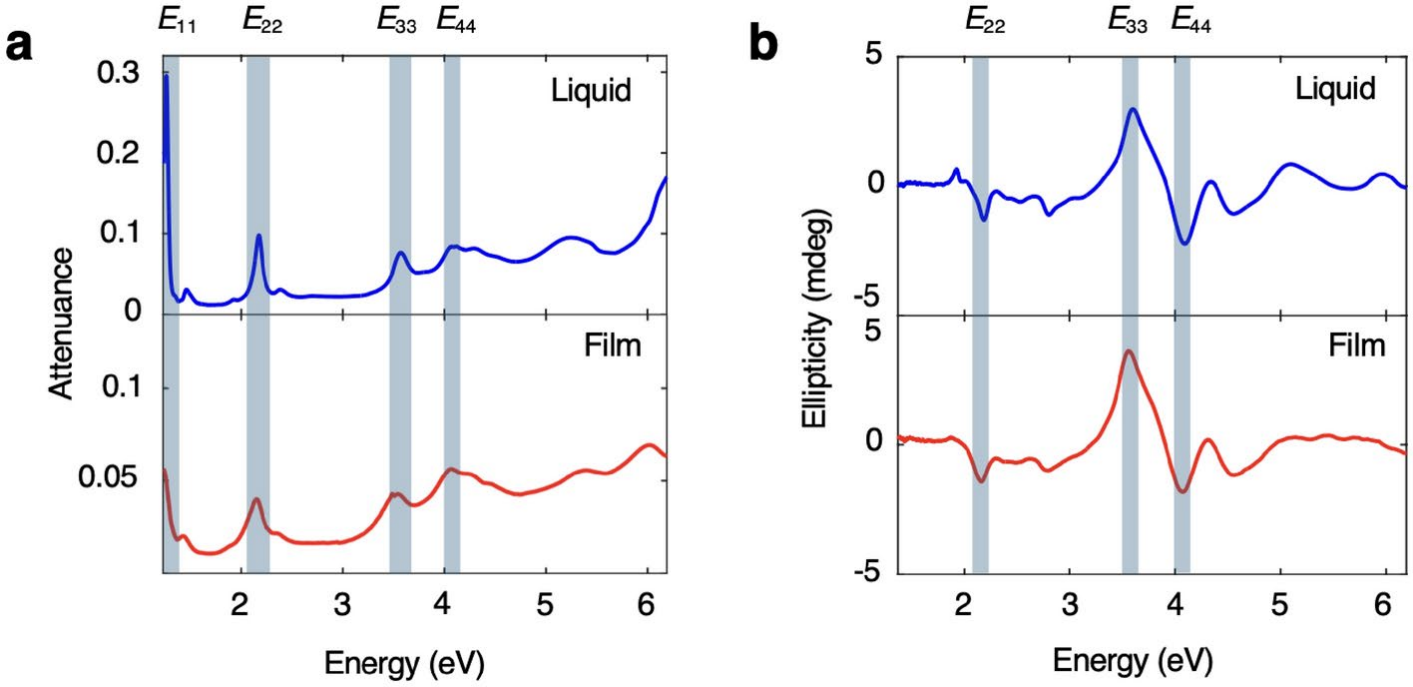
Stationary cuvette demonstration. Comparison of (**a**) UV–VIS attenuation and (**b**) CD spectra for enantiomer-pure single-chirality (6,5) CNTs in liquid and film form. Peaks arising from exciton transitions are labeled and highlighted in blue. $E_{11}$ ($<1.2$ eV), $E_{22}$ (2.2 eV), $E_{33}$ (3.5 eV), and $E_{44}$ (4.1 eV) transition peaks are labeled in both the liquid and film UV–VIS spectra (**a**). The $E_{22}$ (2.2 eV), $E_{33}$ (3.5 eV), and $E_{44}$ peaks (4.1 eV) are labeled in both the liquid and film CD spectra and the sign of the CD signal alternates between adjacent exciton peaks (**b**).

Figure 3 shows attenuation and ellipticity spectra for an aqueous suspension of CNTs contained in a standard cuvette measured in a UV–VIS spectrophotometer and a CNT film measured in the same instrument using the stationary cuvette. All spectra in Fig. 3 are from samples made from left-handed enantiomer-pure single-chirality (6,5) semiconducting CNTs^16^.

In the attenuation spectra, we observe peaks resulting from the $E_{11}$ ($<1.2$ eV), $E_{22}$ (2.2 eV), $E_{33}$ (3.5 eV), $E_{44}$ (4.1 eV) exciton transitions in both the liquid and film samples, distinctive absorbance features of (6,5) carbon nanotubes^17,18^. Similarly, in the CD spectra the $E_{22}$, $E_{33}$, and $E_{44}$ peaks are identifiable in both the liquid and film spectra. We see a slight redshift and change in attenuation magnitude from the liquid to the film samples due to a change in environments for the excitons^19^. The correspondence between peaks in the liquid and film spectra demonstrates the cuvette’s ability to enable the accurate measurement of two forms of the same species using a single instrument.

**Fig. 4:**
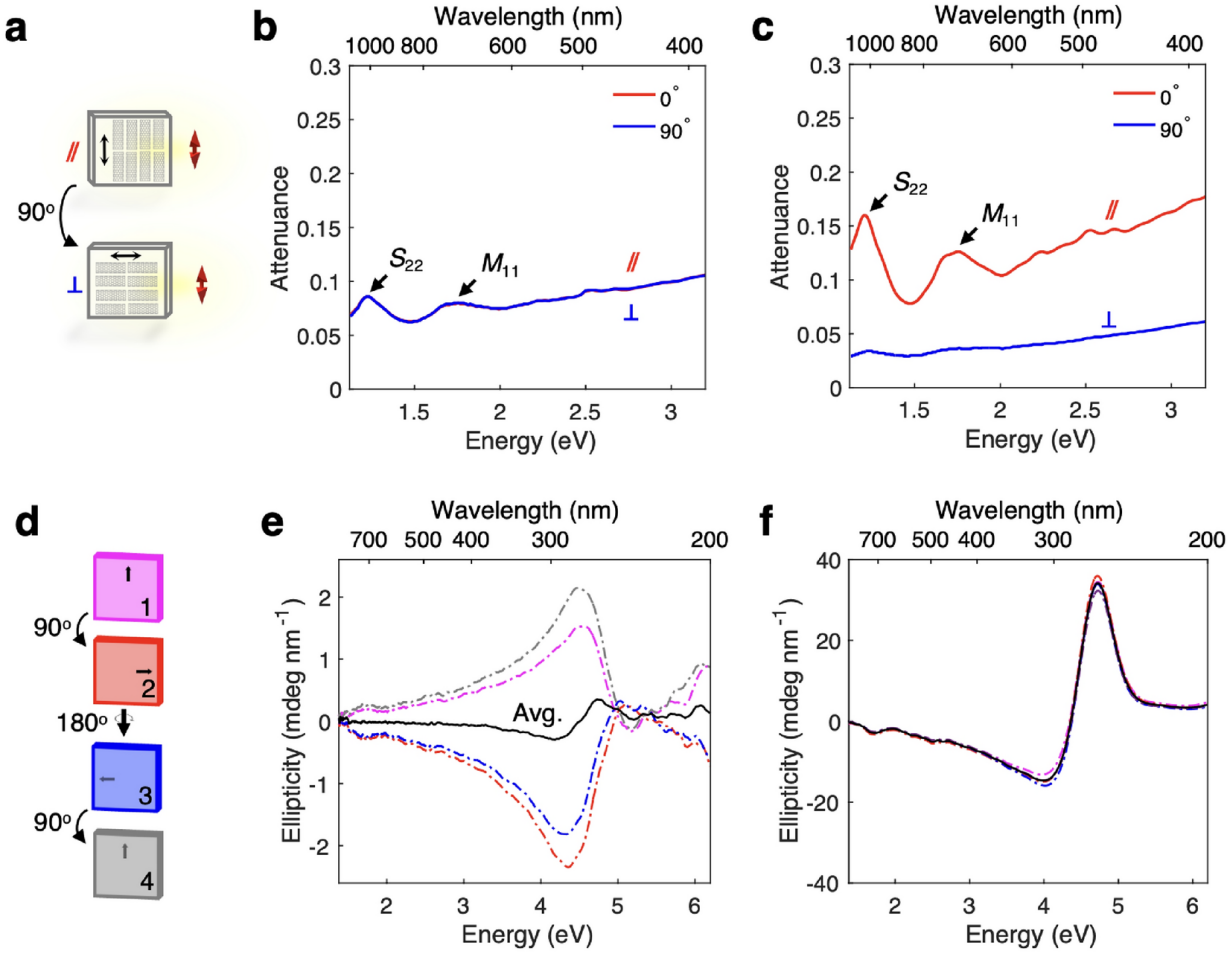
Rotating cuvette demonstration. (**a**) CNT film attenuation spectra were measured at two perpendicular orientations. In an aligned film, spectra were measured with the nanotube axis oriented parallel and perpendicular to incident light polarization. (**b,c**) Polarization-dependent optical attenuation spectra for an unaligned, isotropic (**b**) and aligned, anisotropic (**c**) CNT film from the near-infrared to deep ultraviolet. In (**c**) the red line is for the parallel orientation and the blue line is for the perpendicular orientation. Peaks resulting from interband transitions from semiconducting and metallic CNTs ($S_{22}$ and $M_{11}$, respectively) are labeled in both graphs. (**d**) A four-configuration measurement technique was used to isolate the CD signals. Spectra were measured in plane $0^\circ$ and $90^\circ$, and out of plane $90^\circ$ and $180^\circ$. (**e,f**) Individual and average ellipticity spectra for an unaligned (achiral) CNT film (**e**) and an aligned and twisted (chiral) CNT film (**f**). Red, blue, magenta, and grey lines are spectra measured at a single orientation and black lines show the average over all orientations, or the true CD of the material.

In Fig. 4, we demonstrate the functionality of the rotating cuvette for both UV–VIS and CD measurements. We used the rotating cuvette to obtain polarization-dependent attenuation spectra for unaligned and aligned CNT films. In the isotropic film (Fig. 4b), we observed no difference between spectra measured at the parallel and perpendicular orientations, as expected for an isotropic material. However, in the aligned film (Fig. 4c), there is a strong polarization dependence. When the polarization of incident light is parallel to the nanotube axis (shown in red), strong absorption occurs through transitions between subbands with the same index. We can see the $E_{22}$ transition of semiconducting CNTs at 1.2 eV, labeled S$_{11}$, and the $E_{11}$ transition of metallic CNTs at 1.7 eV, labeled M$_{11}$^20,21^. In contrast, when the film is oriented perpendicular to the polarization of incident light (shown in blue), absorption occurs through transitions between subbands whose indices differ by 1, and their intensities are suppressed because of the depolarization effect^22^, resulting in negligible absorbance^23^. These measurements of a solid sample at different orientations allow us to estimate the degree of alignment in a CNT film^17^, one example of the rotating cuvette’s utility.

We also demonstrate the effectiveness of our rotating cuvette in CD measurements in Fig. 4. CD values influenced by signals that do not arise from true spatial symmetry breaking are often reported in the literature^24^. For these measurements, we used the rotating cuvette to enable measurement of the same portion of the sample at four different orientations, allowing us to isolate the true CD signal of the CNT films^25,26^. More specifically, the measured CD can be expressed as

1 $${\text{C}}{{\text{D}}_{{\text{measured}}}} = {\text{CD}} + \frac{{{\text{LB}} \cdot {\text{ }}LD' + {\text{ }}LB \cdot {\text{LD}}}}{2} + (LD'\sin 2\theta  - {\text{LD}}\cos 2\theta )\sin \alpha ,$$

where CD, LB, and LD are the true CD, linear birefringence, and linear dichroism contributions, respectively, $LB'$ and $LD'$ denote the contributions of LD and LB at 45$^\circ$ rotation, $\theta$ is the in-plane rotation angle of the sample, and $\alpha$ is the residual static birefringence of the modulator. Averaging data with four configurations, offset by 90 degrees, will cancel the second and third terms, giving us the true CD of the material^25^.

In Fig. 4e, we show the four configurations at which measurements were taken to isolate the CD signal (in plane $0^\circ$ and $90^\circ$, out of plane $90^\circ$ and $180^\circ$). Figure 4e shows four-configuration CD measurements for an achiral, unaligned CNT film. Red, blue, magenta, and gray lines show ellipticity measurements taken at a single orientation, while the black line shows the average over all 4 measurement configurations, or the true CD signal. Notably, measurement at only a single orientation would give a significant signal due to the effects of LD and LB while the true CD signal is negligible. In Fig. 4f, we display CD data for a set of two aligned CNT films stacked at an angle, which have been shown to exhibit large CD in the UV region^26^. As expected, we observe a large CD signal, demonstrating the cuvette’s effectiveness in chiral materials. Thus, the cuvette’s ability to rotate $360^\circ$ makes it a reliable tool for measuring CD of solid samples without influence from signals that do not arise from spatial symmetry breaking, a common shortcoming of solid sample CD measurements reported in the literature^24^.

## Conclusions

We designed and fabricated two 3D-printed cuvettes for measuring solid sample in any commercial spectroscopy systems that are designed for liquid samples, eliminating the need to purchase commercial accessories or other spectrophotometers to analyze solid samples. Both cuvettes can be replicated with SLA or FDM 3D printing and can be modified for use for any solid material and with any instrument. We present accurate UV–VIS spectra of solid samples measured with liquid-sample UV–VIS spectrophotometers with the stationary cuvette, and demonstrated the utility of the rotating cuvette in CD spectroscopy of solid samples. In both cases, the cuvettes eliminated the need to buy commercial accessories or construct complex homemade setups.

The results of this work demonstrate the potential for these cuvettes in spectroscopy as low-cost, 3D-printable, and versatile alternatives to existing solid-sample adapters.

## Methods

Aqueous suspensions of single-wall CNTs (SWCNTs) synthesized by the Arc Discharge method (P2-SWNT) purchased from Carbon Solutions, Inc., were used for measurements in Fig. 4. The average length and diameter values were 240 nm and 1.44 nm, respectively. The CNTs were a mixture of semiconducting and metallic species, with a ratio of 2:1. CoMoCAT SWCNTs (704148, Sigma-Aldrich) were used for measurements in Fig. 3. We purified (6,5)- CNT species with gel chromatography^27^.

CNT films were prepared by the controlled vacuum filtration method^28,29^. First, the concentration of sodium deoxycholate (DOC) in the CNT solution was reduced from 0.3% to 0.075% by diluting with pure water before filtration. A total of 4 mL of this solution was filtered through a Whatman Nuclepore Track-Etched membrane. The filtration process included gravitational, pumping, and drying phases to control the nematic ordering of the nanotubes. After filtration, the film was transferred onto a fused silica substrate by dissolving the polycarbonate membrane with chloroform, cleaning with isopropanol (IPA), and drying with air. More information can be found in^28,30^.

For Fig. 4, stacks of aligned films were created using a twist stacking method, where one aligned film is transferred unto a substrate, and another film is transferred on top of the first at a specific angle^26^.

All attenuance spectra were taken with a Cecil 2041 UV–Vis Spectrophotometer. For the solid sample attenuance spectra in Figs. 3, 4b,c, we placed a polarizer between the beam emitter and the sample. All CD data was taken with a JASCO J-815 CD Spectropolarimeter.

## Supplementary Information

Supplementary Information.

## Data Availability

All data is provided within the manuscript or supplementary information files.
