# Supplementary material for: Enabling solid sample analysis in liquid spectrophotometers with a 3D-printed cuvette
Source: Sci Rep. 2025 Feb 20;15:6228. doi: 10.1038/s41598-025-88611-2 (PMC11842853; doi:10.1038/s41598-025-88611-2)
Supplement: Supplementary file 1 — Supplementary Information. [file 41598_2025_88611_MOESM1_ESM.pdf]

Supplementary Information: Fabrication of 3D printed cuvettes for solid sample analysis

Jacques Doumani[1,2], Henry Mansfield[1], Andrey Baydin[1,3,4], Somesh Sasmal[5], Mario El Tahchi[6,7], Weilu Gao[8] and Junichiro Kono[1,3,4,5,9,10]

[1]Department of Electrical and Computer Engineering, Rice University, Houston, TX 77005, USA.
[2]Applied Physics Graduate Program, Smalley-Curl Institute, Rice University, Houston, Texas 77005, USA.
[3]Rice Advanced Materials Institute, Rice University, Houston, TX 77005, USA.
[4]Smalley-Curl Institute, Rice University, Houston, Texas 77005, USA.
[5]Department of Materials Science and NanoEngineering, Rice University, Houston, Texas 77005, USA.
[6]Department of Physics, Lebanese University, Jdeidet, Lebanon, 90656.
[7]Laboratory of Biomaterials and Intelligent Materials, Lebanese University, Jdeidet, Lebanon, 90656.
[8]Department of Electrical and Computer Engineering, The University of Utah, Salt Lake City, Utah 84112, USA.
[9]Carbon Hub, Rice University, Houston, Texas 77005, USA.
[10]Department of Physics and Astronomy, Rice University, Houston, Texas 77005, USA.

Contributing authors: kono@rice.edu;

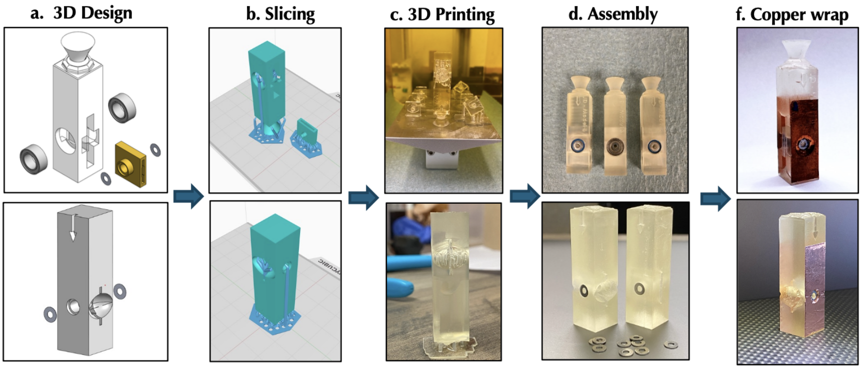

Fig. 1 **Cuvette Fabrication** All images in the figure are of SLA printed cuvettes. The same process, excluding any exceptions mentioned below, was used to print FDM cuvettes. In the figure top images are of the rotating cuvette and bottom images are of the stationary cuvette. (a) Cuvettes were designed using SolidWorks. (b) SLA printed cuvettes were sliced and printed using Anycubic Photon Slicer software. FDM printed cuvettes were sliced and printed using Cura software. SLA and FDM slicing parameters are listed in Supplementary Notes 1 and 2 respectively. (c) All cuvettes and pockets were printed with supports in a bottom face down configuration. Cuvettes needed to be cleaned and bath sonicated before assembly. (d) Aperture systems were assembled after cleaning and sonication. SLA printed parts needed to be UV cured (not shown) to prevent deformation during use. (f) Copper tape was applied to the sides of cuvettes with aperture openings before use.

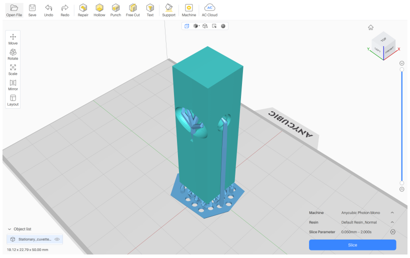

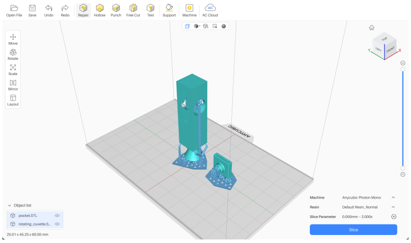

Supplementary Note 1: SLA Printing Parameters SLA prints were controlled using Anycubic Photon Workshop. Supports were used for all parts. Layer thicknesses of 0.05 - 0.1mm, normal exposure times of 2.5 - 3 seconds, off time of 0 - 0.5 seconds, bottom exposure time of 30 seconds, and 5 - 6 bottom layers were used. Basic control mode with a z lift distance of 6mm, a z lift speed of 1-4mm/s, and z retract speeds of 5-6mm/s were used. Anti alias was set to 1.

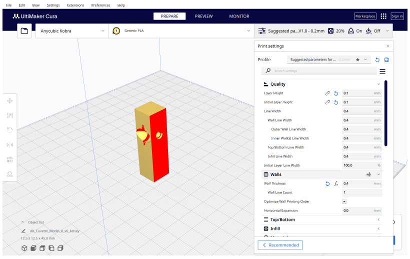

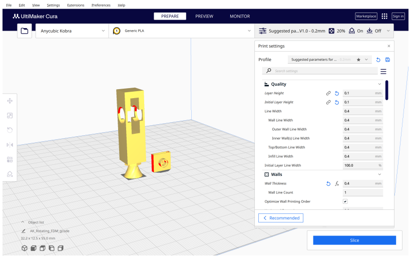

Supplementary Note 2: FDM 3D Printing Parameters Prints were controlled using the Cura slicing software. Prints were performed using a nozzle temperature of 220°C, a bed temperature of 60°C, a layer height of 0.1 mm and a wall thickness of 0.4 mm. A print speed of 35 mm/s was used. Supports were used on all prints. Support overhang angle of 0.0°, support density of 0.5%, and horizontal expansion of 0.0 mm were used. Build plate adhesion type was set to skirt. Suggested parameters for "PLA_V1.0 - 0.2mm" were used for all parameters not specified above.

Table 1 Equipment and Materials

Equipment	Brand
SLA 3D Printer	Anycubic Photon Mono
FDM 3D Printer	Anycubic Kobra
SLA Resin	Esun Water Washable
FDM Filament	Anycubic PLA Filament
Washers	Xike 623-2RS 3x10x4mm
Bearings	M2 Stainless Steel Flat Washer
Curing Station	Elegoo Mercury Plus v1.0

Table 2 Cuvette Use A traditional cuvette, the stationary cuvette, and the rotating cuvette in use in a JASCO J15 Spectrophotometer

Liquid Cuvette	Stationary Cuvette	Rotating Cuvette
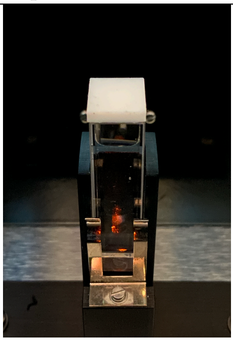	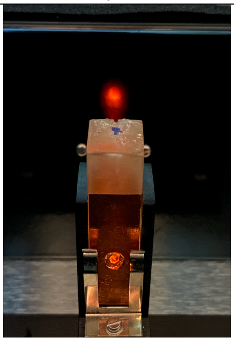	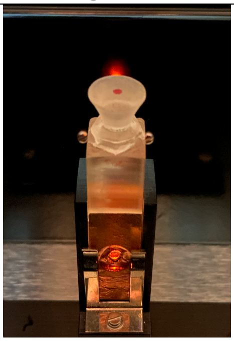
